# S15-1: Sports clubs as health promotion settings: From physical activity to sustainable sport - a question of how!

**DOI:** 10.1093/eurpub/ckae114.265

**Published:** 2024-09-26

**Authors:** Susanna Geidne, Helena Ericson, Mikael Quennerstedt, Aurélie Van Hoye

**Affiliations:** Örebro University, Sweden; Örebro University, Sweden; Örebro University, Sweden; Örebro University, Sweden

## Abstract

**Purpose:**

The concepts of public health, health and health promotion have been used for decades in relation to sport in policy, research and practice. That sport has some kind of significance for and connection to public health is clear. But how can we understand the relationships between sport and public health?

The aim of this presentation is to argue that what is regarded as knowledge about the relationship between sport and public health largely depends on how the concepts of public health, health, health promotion and sport are framed.

**Methods:**

In this presentation, we theorize the relationship between sport and public health as different patterns and give examples of how sports clubs can thus act as a health-promoting setting for different target groups.

**Results:**

A first attempt to categorize the patterns in the four categories (i) health promotion as an outcome of sport, (ii) health promotion through sport, (iii) health promotion in sport, and (iv) health promoting sport. In these four categories we reveal research, that because of how they define health promotion and sport, are quite different. The different categories also use the concept of sport differently, as something that will always be healthy because it contains physical activity, as a mean, as an arena where you reach people or in a more integrated way.

**Conclusions:**

We argue that it is not enough to recognize that physical activity and sport are good for people's physical health. By using a more inclusive conceptualization of how health can be promoted in, through and as health-promoting sport, health promotion studies can feed into the conclusions of other studies, i.e. what can be done about the how-question. What we propose and provide empirical examples of in this presentation can be seen as a theoretical basis for discussing the relationship or relationships between sport and public health that can be used in policy, research and practice. It is therefore possible to broaden the issue, to make it more complex, to go beyond compartmentalized thinking and to contribute to sustainable change in sport. Because, sustainable sport can be a question of how!

**Support/Funding Source:**

None

